# The Effect of Phloroglucinol, A Component of *Ecklonia cava* Extract, on Hepatic Glucose Production

**DOI:** 10.3390/md15040106

**Published:** 2017-04-05

**Authors:** Ji-Young Yoon, Hojung Choi, Hee-Sook Jun

**Affiliations:** 1 College of Pharmacy, Gachon Institute of Pharmaceutical Science, Gachon University, Yeonsu-gu, Incheon 21936, Korea; yoonj6@naver.com (J.-Y.Y.); hchoi@gachon.ac.kr (H.C.); 2 Lee Gil Ya Cancer and Diabetes Institute, Gachon University, Yeonsu-gu, Incheon 21999, Korea; 3 Gachon Medical Research Institute, Gil Hospital, Namdong-gu, Incheon 21565, Korea

**Keywords:** Phloroglucinol, gluconeogenesis, PEPCK, G6Pase, AMPKα

## Abstract

Phloroglucinol is a phenolic compound that is one of the major compounds in *Ecklonia cava* (brown alga). It has many pharmacological activities, but its anti-diabetic effect is not yet fully explored. In this study, we investigated the effect of phloroglucinol on the control of blood glucose levels and the regulation of hepatic glucose production. Phloroglucinol significantly improved glucose tolerance in male C57BL/6J mice fed a high fat diet (HFD) and inhibited glucose production in mouse primary hepatocytes. The expression of phosphoenol pyruvate carboxykinase (PEPCK) and glucose-6-phosphatase mRNA and protein (G6Pase), enzymes involved in gluconeogenesis, were inhibited in liver tissue from phloroglucinol-treated mice and in phloroglucinol-treated HepG2 cells. In addition, phloroglucinol treatment increased phosphorylated AMP-activated protein kinase (AMPK)α in HepG2 cells. Treatment with compound C, an AMPKα inhibitor, inhibited the increase of phosphorylated AMPKα and the decrease of PEPCK and G6Pase expression caused by phloroglucinol treatment. We conclude that phloroglucinol may inhibit hepatic gluconeogenesis via modulating the AMPKα signaling pathway, and thus lower blood glucose levels.

## 1. Introduction

Hepatic glucose production is a key physiological process that becomes altered in diabetic patients and represents the main target of the anti-hyperglycemic effect of biguanides [1,2]. Patients with poorly controlled type 2 diabetes have increased rates of endogenous glucose production. The liver can produce glucose by breaking down glycogen (glycogenolysis) and by de novo synthesis of glucose from lactate, pyruvate, glycerol, and alanine (gluconeogenesis) [3,4]. Therefore, the regulation of hepatic glucose production can contribute to the reduction of blood glucose levels in diabetes [5].

*Ecklonia cava* (*E. cava*), an edible brown alga, is found abundantly in the oceans of Korea and Japan [6]. *E. cava* extract exhibits free radical scavenging activity, anti-plasmin inhibiting activity, antimutagenic activity, and bactericidal activity [7,8,9]. *E. cava* extract reduces blood glucose levels and increases insulin levels in streptozotocin-induced diabetic mice, a model of type 1 diabetes [10], and the dieckol-rich extract of *E. cava* improves glucose and lipid metabolism in C57BL/KsJ-db/db mice, a model of type 2 diabetes [11]. In addition, the extract of *E. cava* produced from the Gijang area in Korea is more effective for weight loss and reducing hyperglycemia in high fat diet (HFD)-induced obese mice compared to the extract of *E. cava* from the Jeju area in Korea [12,13]. Further study showed that Gijang *E. cava* extract has higher concentrations of phloroglucinol than Jeju *E. cava* extract [12].

*E. cava* extract contains abundant phlorotannin compounds including phloroglucinol, eckol, dieckol, triphlorethol A, and eckstolonol [7,8]. Phloroglucinol is a member of the group of organic compounds known as polyphenols and is one of the phlorotannins that is also present at high levels in *Pseudomonas*, *Ishige okamurae*, *Garcinia subelliptica*, *Fucaceae* as well as *E. cava* [14]. Phloroglucinol has a wide range of applications in the pharmaceutical, cosmetic, textile, paint, and dyeing industries [15], and is widely used as a treatment of gastrointestinal disorders such as gallstones or spasmodic pain [16]. In addition, phloroglucinol has broad therapeutic effects, including anti-inflammatory, anti-microbial, anti-allergy, anti-oxidant, and anti-cancer effects [14,17,18,19]. However, the anti-diabetic effect of phloroglucinol has not been studied. 

In this study, we investigated the effect of phloroglucinol on blood glucose control and hepatic glucose production. Phloroglucinol treatment improved glucose tolerance in mice fed an HFD and decreased the expression of gluconeogenic enzymes such as phosphoenol pyruvate carboxykinase (PEPCK) and glucose-6-phosphatase (G6Pase) in liver tissue. Phloroglucinol inhibited glucose production in hepatocytes, and studies on the mechanisms revealed that phloroglucinol decreased PEPCK and G6Pase gene expression via AMPKα activation, subsequently inhibiting hepatic glucose production.

## 2. Results

### 2.1. Administration of Phloroglucinol Improved Glucose Tolerance and Reduced PEPCK and G6Pase Expression Levels in HFD-Induced Obese Mice

**Figure 1:**
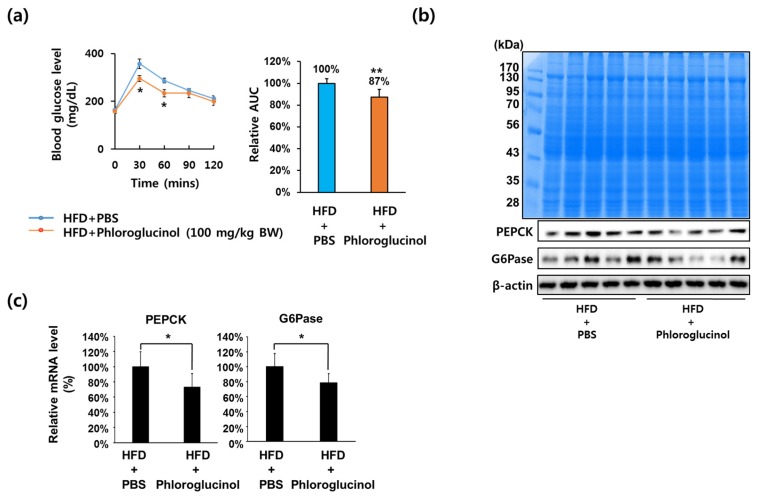
Effects of phloroglucinol on PEPCK and G6Pase gene expression in high fat diet (HFD)-induced obese mice. Ten weeks after beginning an HFD, C57BL/6J mice were orally administered with 100 mg/kg body weight of phloroglucinol (HFD + phloroglucinol) or phosphate-buffered saline (PBS) (HFD + PBS) daily for 9 weeks. (**a**) Oral glucose tolerance tests were performed at 8 weeks after administration of phloroglucinol. Relative area under curve (AUC) was measured. Liver tissue was lysed to obtain protein and mRNA. (**b**) Protein bands were detected by Coomassie blue staining (upper) and protein levels of PEPCK, G6Pase and β-actin were analyzed by Western blot (lower) and (**c**) mRNA levels of PEPCK and G6Pase were analyzed by RT-qPCR. Data are presented as means ± standard deviation (SD) *n* = 5/group (* *p* < 0.05 vs. HFD-PBS group; ** *p* < 0.01 vs. HFD-PBS group; Student’s *t*-test).

To address whether phloroglucinol has an effect on blood glucose levels, six-week-old C57BL6 male mice were fed an HFD for 10 weeks and then orally administered 100 mg/kg of phloroglucinol daily for 9 weeks. As shown in Figure 1A, oral glucose tolerance tests after 8 weeks of treatment showed that blood glucose levels in the HFD + phloroglucinol group were significantly decreased at 30 and 60 mins following 2 g/kg of oral administration of glucose compared with the HFD + phosphate-buffered saline (PBS) group. In order to investigate whether phloroglucinol treatment affects PEPCK and G6Pase gene expression in the liver, we performed Western blotting analysis for PEPCK and G6Pase protein expression in liver tissue of the HFD + phloroglucinol and HFD + PBS mice after 9 weeks of treatment. As shown in Figure 1B, the expression of PEPCK and G6Pase protein was decreased in the HFD + phloroglucinol group compared with the HFD + PBS group. RT-qPCR analysis also showed that PEPCK and G6Pase mRNA levels were decreased by the administration of phloroglucinol (Figure 1C).

### 2.2. Phloroglucinol Reduced the Glucose Production in Mouse Primary Hepatocyte

**Figure 2:**
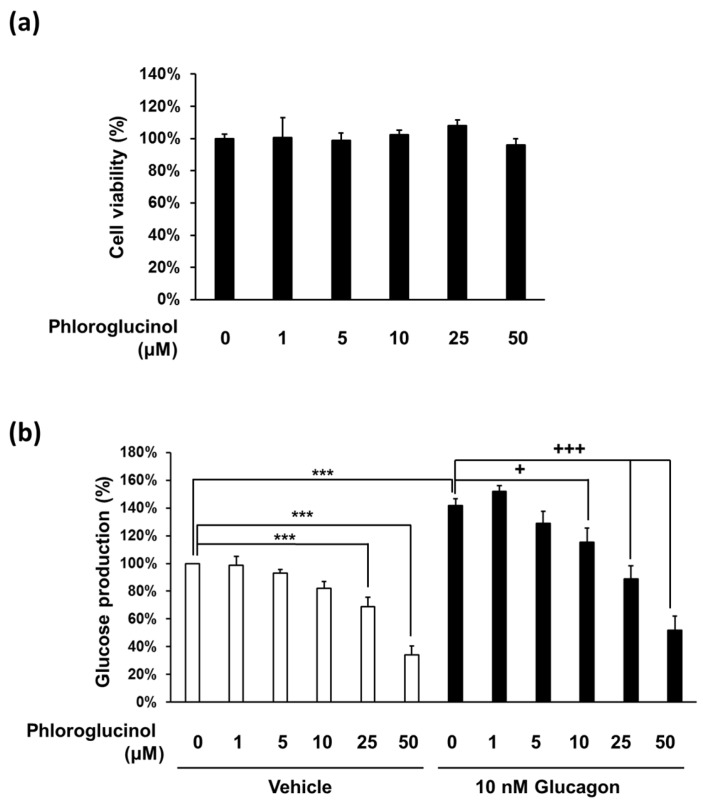
Effects of phloroglucinol on glucose production in mouse primary hepatocytes. (**a**) Mouse primary hepatocytes were treated with the indicated concentrations of phloroglucinol for 24 h and cell viability was determined by CCK-8 assay. Data are presented as means ± standard deviation (SD), three independent experiments were performed; (**b**) Mouse primary hepatocytes were treated with the indicated concentrations of phloroglucinol in the absence or presence of 10 nM glucagon and gluconeogenic substrates (2 mM pyruvate and 20 mM sodium lactate). Glucose production was measured by a glucose assay kit. Data are presented as means ± standard deviation (SD), five independent experiments were performed. (*** *p* < 0.001 vs. Vehicle, + *p* < 0.05 or ++ *p* < 0.01 vs. 10 nM glucagon; one-way ANOVA).

To investigate the effect of phloroglucinol on hepatic glucose production, we used primary hepatocytes rather than the hepatoma cell line, because it is not easy to obtain consistent results using the hepatoma cell line. We first checked whether phloroglucinol has any cytotoxic effects on isolated mouse primary hepatocytes. The cell viability observed after treatment with phloroglucinol up to 50 μM for 24 h was similar to that of the control without phloroglucinol treatment in mouse primary hepatocytes (Figure 2A). We examined glucose production in mouse primary hepatocytes treated with different concentrations of phloroglucinol (1–50 μM) for 3 h. Glucose production began to decrease with 5 μM phloroglucinol and decreased further in a dose-dependent manner. In addition, glucagon-induced glucose production was similarly decreased beginning at 10 μM concentration in a dose-dependent manner (Figure 2B).

### 2.3. Phloroglucinol Decreased PEPCK and G6Pase Gene and Protein Expression in HepG2 Cells

**Figure 3:**
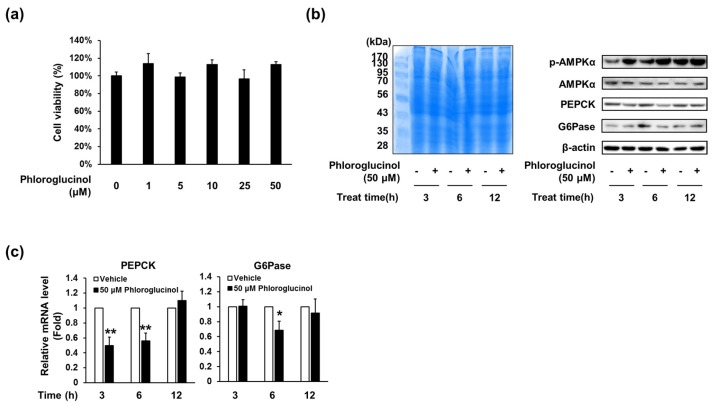
Effects of phloroglucinol on PEPCK and G6Pase gene expression levels in HepG2 cells. (**a**) HepG2 cells were treated with the indicated concentrations of phloroglucinol for 24 h and cell viability was determined by CCK-8 assay. HepG2 cells were treated with 50 μM phloroglucinol for the indicated times; (**b**) Protein bands were detected by Coomassie blue staining (left) and phospho-AMPKα (Thr-172), AMPKα, PEPCK and G6Pase protein levels were detected by Western blotting (right) and (**c**) PEPCK and G6Pase mRNA expression levels were analyzed by RT-qPCR. Data are presented as means ± standard deviation (SD), three independent experiments were performed. (* *p* < 0.05 and ** *p* < 0.005 vs. vehicle; Student’s *t*-test)

As phloroglucinol decreased glucose production in primary hepatocytes, we investigated whether phloroglucinol affects the expression of enzymes for gluconeogenesis, such as PEPCK and G6Pase, in HepG2 cells. Cell viability assay showed that phloroglucinol treatment did not affect the viability of HepG2 cells (Figure 3A). Western blot analysis showed that the expression of PEPCK and G6Pase protein was decreased from 3 or 6 h of treatment with 50 μM of phloroglucinol compared with the control group (Figure 3B). Phosphorylated AMPK was also increased by phloroglucinol treatment. In addition, PEPCK mRNA level was decreased after 3 and 6 h of treatment and G6Pase mRNA level was decreased after 6 h of treatment (Figure 3C).

### 2.4. Decrease of PEPCK and G6Pase Gene Expressions by Phloroglucinol in HepG2 Cells Was Mediated by AMPKα Activation

**Figure 4:**
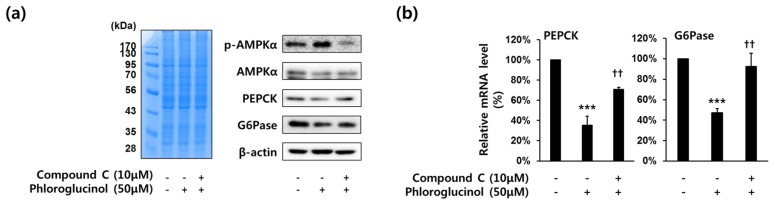
Effects of compound C (AMPKα inhibitor) on phloroglucinol-induced suppression of PEPCK and G6Pase gene expression in HepG2 cells. Cells were treated with 10 μM of compound C and 50 μM of phloroglucinol for 6 h in high glucose DMEM. (**a**) Protein bands were detected by Coomassie blue staining (left) and phospho-AMPKα (Thr-172), AMPKα, PEPCK and G6Pase protein levels were analyzed by Western blotting (right) and (**b**) PEPCK and G6Pase mRNA expression levels were analyzed by RT-qPCR. Data are presented as means ± standard deviation (SD), three independent experiments were performed. (*** *p* < 0.001 vs. vehicle or ^††^ *p* < 0.005 vs. phloroglucinol; Student’s *t*-test).

We investigated whether phloroglucinol inhibited PEPCK and G6Pase gene expression by the stimulation of AMPKα phosphorylation in HepG2 cells, because activated AMPKα is known to inhibit gluconeogenesis [20]. To test whether the expression of PEPCK and G6Pase gene is affected by the treatment of AMPKα inhibitor (Compound C), HepG2 cells were treated with 50 μM phloroglucinol and 10 μM compound C for 6 h. As shown in Figure 4A, phosphorylation of AMPKα was increased by phloroglucinol treatment and PEPCK and G6Pase protein expression were decreased by phloroglucinol treatment, but compound C treatment inhibited this effect of phloroglucinol. Also, we performed RT-qPCR to determine the expression of PEPCK and G6Pase mRNA levels. The decreased expression of PEPCK and G6Pase mRNA levels by treatment with phloroglucinol was reversed by treatment with compound C (Figure 4B).

## 3. Discussion

Many kinds of compounds derived from marine organisms have been tested as possible nutraceutical or medical materials [21,22,23]. In particular, substances isolated from algae are attracting attention as pharmaceuticals [22,24,25]. For instance, extracts of *Callophyllis japonica*, *Gracilaria tenuistipitata,* and *Bryothamnion triquetrum*, red algae, show antioxidant, anti-nociceptive and anti-inflammatory effects in mouse models [26,27,28]. In addition, bis-2,3-dibromo-4,5-dihydroxybenzyl ether, a compound isolated from *Odonthaliacorymbifera*, another red alga, shows anti-diabetic activity [29]. A methanol extract of *Chaetomorpha antennina,* a green alga, inhibits the activity of key diabetic enzymes, including α-glucosidase, α-amylase, and dipeptidyl peptidase-IV [30]. In *Ecklonia* species, brown algae, the functional components are polyphenols, known as phlorotanins, including dieckol, phlorogucofuroeckol A, 7-phloroeckol, and octaphlorethol A. These compounds show anti-diabetic activities by the regulation of α-glucosidase activity, glucose uptake in skeletal muscle, protein tyrosine phosphatase 1B enzyme activity, and AMPKα activity [7,21,31].

The extract of *E. cava*, a brown alga, has potential as a therapeutic agent for diabetes, evidenced by several previous studies [10,12,13,32] and its effects on reducing blood glucose levels in HFD-induced diabetic mice [12,13]. The therapeutic effect of *E. cava* extract in diabetic models might be, at least in part, due to the effect of phloroglucinol, which is a major component of *E. cava* extract. Recently it was reported that phloroglucinol showed protective effects on the glucotoxicity-induced apoptosis of beta-cells [33]. We first examined the effect of phloroglucinol on blood glucose control and found that oral administration of phloroglucinol significantly improved glucose tolerance in HFD-fed mice. 

As it was previously reported that phloroglucinol from *Mukia maderspatana* inhibits hepatic glucose production in rat primary hepatocytes [34], we then examined whether phloroglucinol has a similar effect on glucose production in mouse primary hepatocytes. Phloroglucinol treatment reduced both basal glucose production and glucagon-induced glucose production in a dose-dependent manner. These results indicate that the improvement of glucose tolerance might be due to the inhibition of glucose production from hepatocytes.

Gluconeogenesis is generally regulated by gluconeogenic enzymes such as PEPCK and G6Pase in the liver. Therefore, we checked the expression of these enzymes in HepG2 cells treated with phloroglucinol. We found that phloroglucinol treatment decreased PEPCK and G6Pase mRNA and protein expression in HepG2 cells. These data mean that phloroglucinol inhibits glucose production by decreasing PEPCK and G6Pase expression. 

AMPK is a central regulator of energy metabolism, and the activation of hepatic AMPK plays an important role in the inhibition of glucose production in the liver [20,35]. PEPCK and G6Pase gene expression is regulated by several transcription factors such as cAMP response element binding protein (CREB), CREB-regulated transcription coactivator 2 (CRTC2;TORC2), forkhead box O1 (FOXO1), peroxisome proliferator-activated receptor gamma coactivator-1 alpha (PGC-1α), and hepatocyte nuclear factor 4 alpha (HNF4α) [20,35]. It is known that AMPKα phosphorylates CRTC2 and subsequently inhibits the expression of gluconeogenic genes [36]. We found that AMPKα phosphorylation was increased by the treatment of 50 μM phloroglucinol for 6 hours in HepG2 cells. Consistent with this result, previous reports showed that phloroglucinol-rich *E. cava* extract activates AMPKα by phosphorylation and decreases Akt phosphorylation [10,12,13]. 

In order to investigate whether the effect of phloroglucinol on the downregulation of PEPCK and G6Pase is mediated by the AMPKα signaling pathway, we conducted experiments using compound C, which is an inhibitor of AMPKα [20]. Compound C treatment inhibited the induction of AMPKα phosphorylation and the reduction of gene expression of PEPCK and G6Pase by phloroglucinol treatment. These data strongly support that phloroglucinol decreases gene expression of PEPCK and G6Pase through the activation of the AMPKα signaling pathway.

## 4. Materials and Methods

### 4.1. Materials

Phloroglucinol and glucagon were purchased from Sigma-Aldrich (St. Louis, MO, USA) and dissolved in 0.1% DMSO and distilled water, respectively. Antibodies against AMPKα and phospho-AMPKα (Thr-172) were purchased from Cell Signaling Technology (Beverly, MA, USA) and anti-PEPCK and β-actin were purchased from Santa Cruz Biotechnology (Santa Cruz, CA, USA). Anti-G6Pase was purchased from Abcam (Cambridge, MA, USA). Compound C and glucose-free DMEM were purchased from Sigma-Aldrich (St. Louis, MO, USA).

### 4.2. Animals and Administration of Phloroglucinol to HFD-Induced Obese Mice

C57BL/6J and C57BL/6N male mice were obtained from OrientBio (Kyounggido, Korea). Mice were housed at the animal facility of the Lee Gil Ya Cancer and Diabetes Institute (CACU, Gachon University, Incheon, Korea), in accordance with “Guidelines and Animal for Users”. Six week-old male C57BL/6J mice were fed an HFD (60% fat primarily from lard, Research Diets, Inc., New Brunswick, NJ, USA, #D12492). After 10 weeks (at 16 weeks of age), mice were randomly divided into two groups: the PBS-treated HFD group (HFD + PBS; *n* = 5) and the phloroglucinol-treated HFD group (HFD + phloroglucinol; *n* = 5). Phloroglucinol (100 mg/kg in PBS) or PBS was administered by oral intubation daily for 9 weeks. Body weight and food consumption were measured weekly.

### 4.3. Glucose Tolerance Tests

Animals were fasted overnight and glucose (2 g/kg) was administrated by oral injection. Blood samples were obtained from tail vein at 0, 30, 60, 90, and 120 min after glucose loading. Blood glucose levels were measured with glucose analyzer (OneTouch^®^ Ultra, Lifescan, Johnson & Johnson, Milpitas, CA, USA).

### 4.4. Isolation of Mouse Primary Hepatocytes

Eight week-old male C57BL/6N mice were anesthetized using 150 μL of ketamine per mouse. The liver was perfused through the portal vein using perfusion buffer I (0.142 M NaCl, 0.0067 M KCl, 0.01 M HEPES, and 2.5 mM EGTA (pH 7.4)) for 10 min and then perfusion buffer II (66.7 mM NaCl, 6.7 mM KCl, 10 mM HEPES, and 0.5 g/L of collagenase (pH 7.6)) for 5 min. The liver was removed and dissected into cold high glucose DMEM to separate the hepatic cells. Cells were centrifuged to 50 g for 5 min at 4 °C, washed using cold high glucose DMEM, and then centrifuged at 50 g for 5 min at 4 °C. Hepatocytes were isolated by percoll gradient centrifugation (45 mL of Percoll, 5 mL of 10X PBS, and 0.5 mL 1M HEPES).

### 4.5. Measurement of Hepatic Glucose Production

Cells were seeded at 2.5 × 10^5^ cells/well in 12-well plates and treated with various concentrations of phloroglucinol for 30 min in glucose-free DMEM. Cells were washed in a pre-warmed glucose-free DMEM medium twice and stimulated with 100 nM of glucagon in the presence of phloroglucinol for another 6 h in glucose-free DMEM. Gluconeogenic substrates, 20 mM sodium lactate and 2 mM sodium pyruvate, were added to cells. Glucose in the media was quantified at 30 min after the addition of substrates using a glucose assay kit and normalized to cellular protein concentrations [20]. Five independent experiments were performed in triplicate.

### 4.6. Cell Culture and Viability Assay

The human hepatoma cell line, HepG2, was purchased from Korean Cell Line Bank (Seoul, Korea). HepG2 cells were grown in DMEM (Welgene Inc, Grand Island, NY, USA) with 10% fetal bovine serum (FBS) and antibiotics (100 unit/mL penicillin and 100 μg/mL streptomycin). Primary mouse hepatocytes were grown in HepatoZYME (GibcoBL, Grand Island, NY, USA) with 10% FSB and 1% antibiotics. Cells were maintained at sub-confluent conditions in a humidified incubator at 37 °C with ambient oxygen and 5% CO_2_. The cytotoxicity of phloroglucinol was determined by Cell Counting Kit-8 assay (Dojindo, Japan). In brief, cells were seeded at 1 × 10^4^ cells/well in 96-well plates and treated with various concentrations of phloroglucinol in HepatoZYME-SFM for 24 h. After one day of treatment, the Cell Counting Kit-8 solution was added and incubated at 37 °C for 2 h. Then the absorbance was recorded at 450 nm using a microplate reader (VersaMax, Molecular Devices, Sunnyvale, CA, USA). Three independent experiments were performed in triplicate.

### 4.7. RT-qPCR Analysis

Total RNA from HepG2 cells and mouse hepatocytes was prepared using the RNAiso reagent (Takara, Otsu, Japan) in accordance with the manufacturer’s protocol. Reverse transcription of 2 μg of total RNA was conducted using the first strand cDNA synthesis kit (Takara, Otsu, Japan) in a reaction volume of 20 μL. Messenger RNA expression was analyzed by quantitative real-time reverse transcription polymerase chain reaction (RT-qPCR) with SYBR green (Takara, Otsu, Japan). All primer sequences used in RT-qPCR experiments are listed in Table 1. Three separate experiments were performed with different samples.

### 4.8. Western Blot Analysis

To detect proteins in whole cell lysates, cells were washed with ice-cold PBS and lysed using a protein extraction kit (GE Healthcare, Piscataway, NJ, USA). Proteins (30 μg) were separated by 10% SDS-PAGE and bands were stained with Coomassie Blue R-250 (ThermoFisher scientific, MA, USA). Proteins were electrophoretically transferred to polyvinylidene difluoride membranes. The transferred membrane was blocked with 5% skim milk for 1 h and incubated with primary antibodies against PEPCK, G6Pase, AMPKα, phospho-AMPKα (Thr-172), or β-actin. Beta-actin was used as a loading control. The membrane was washed three times with TBST (100 mM Tris pH 7.4, 150 mM NaCl, 0.5% Tween-20) for 10 min and incubated with horseradish peroxidase-conjugated goat anti-rabbit IgG (Santa Cruz Biotechnology, Santa Cruz, CA, USA) or goat anti-mouse IgG (Santa Cruz Biotechnology, Santa Cruz, CA, USA) secondary antibodies. Signal was detected using a Fujifilm luminescent mage analyzer LAS4000 with ECL detection kit (Merck Millipore, Darmstadt, Germany). Three or four separate experiments were performed with different samples. 

### 4.9. Statistical Analysis

Comparisons between groups were analyzed using a Student’s *t*-test and one-way ANOVA, where *p* < 0.05 was considered statistically significant. Student’s *t*-test and one way-ANOVA analysis were performed using GraphPad Prism v6.0 (GraphPad Software, Inc., La Jolla, CA, USA). All data are presented as means ± standard deviation (SD) and all experiments were performed three times. 

## 5. Conclusions

We found that phloroglucinol decreased gluconeogenesis by downregulating PEPCK and G6Pase gene expression through AMPKα activation. These findings suggest that phloroglucinol may have a preventive or therapeutic potential for the treatment of diabetes.

## Figures and Tables

**Table 1:** Primer sequences of human mRNA.

Gene Symbol	Sequences	
Human Cyclophilin B	Sense	5′ TGCCATCGCCAAGGAGTAG 3′
	Anti-sense	3′ TGCACAGACGGTCACTCAAA 5′
Human PEPCK	Sense	5′ TGAAAGGCCTGGGGCACAT 3′
	Anti-sense	3′ TTGCTTCAAGGCAAGGATCTCT 5′
Human G6Pase	Sense	5′ TCATCTTGGTGTCCGTGATCG 3′
	Anti-sense	3′ TTTATCAGGGGCACGGAAGTG 5′
